# Impact of Aging and Knee Osteoarthritis on Lower Limb Alignment and CPAK Classification: Gender Differences in a Japanese Cohort

**DOI:** 10.3390/jcm13206250

**Published:** 2024-10-19

**Authors:** Kento Harada, Yu Mori, Masayuki Kamimura, Takashi Aki, Tomoki Koyama, Toshimi Aizawa

**Affiliations:** Department of Orthopaedic Surgery, Tohoku University Graduate School of Medicine, 1-1 Seiryo-machi, Aoba-ku, Sendai 980-8574, Japan; kento.harada.a8@tohoku.ac.jp (K.H.); masayuki.kamimura.b4@tohoku.ac.jp (M.K.); takashi.aki.d6@tohoku.ac.jp (T.A.); tomoki.koyama.e3@tohoku.ac.jp (T.K.); toshimi.aizawa.a5@tohoku.ac.jp (T.A.)

**Keywords:** coronal plane alignment of the knee (CPAK) classification, arithmetic hip-knee-ankle angle (HKA), joint line obliquity, aging, knee osteoarthritis

## Abstract

**Objective:** This study investigates the impact of age and knee osteoarthritis (OA) on the coronal plane alignment of the lower extremity in Japanese males and females, utilizing the Coronal Plane Alignment of the Knee (CPAK) classification system. **Methods:** A cross-sectional analysis was conducted with 150 male and 150 female patients. Participants were divided into three groups according to age and OA progression. The mechanical lateral distal femoral angle (mLDFA) and mechanical medial proximal tibial angle (mMPTA) were measured using standard digital long-leg radiographs. Arithmetic hip-knee-ankle angle (aHKA) and joint line obliquity (JLO) were calculated, and the CPAK classification was performed to verify the distribution among the three groups. **Results:** The results showed increased varus alignment of the mean mLDFA correlated with OA in both genders and with aging in males. The mean mMPTA did not change in males but shifted toward varus in females with both aging and OA. Both genders demonstrated a constitutional varus alignment with the progression of osteoarthritis (males: 1.3 ± 2.4° to −3.5 ± 3.7°, *p* < 0.001; females: −1.2 ± 3.2° to −3.6 ± 2.9°, *p* < 0.001). However, this trend with aging was observed only in females (0.0 ± 2.5° to −1.2 ± 3.2°, *p* = 0.018). JLO maintained its apex distal position with aging and OA progression in all subjects. The study further revealed a notable transition from CPAK Type II to Type I with OA progression in both genders, additionally influenced by aging in females. **Conclusions:** Aging affects coronal alignment and CPAK classification differently across genders. With OA progression, there was a shift toward smaller aHKA, while JLO remained unchanged. Compared to other races, young Japanese people exhibit similar CPAK distributions, but distinct differences appear in OA-affected individuals, highlighting potential racial variations in CPAK classifications.

## 1. Introduction

The variations in the coronal plane alignment of the lower extremity are observed across individuals. They are essential for surgical planning in total knee arthroplasty and around-knee osteotomy [1]. The mechanical lateral distal femoral angle (mLDFA) and the mechanical medial proximal tibial angle (mMPTA) are frequently employed to assess the coronal alignments of the femur and tibia, respectively [2,3], and applied for planning knee surgeries. A report that described the lower limb alignment variation in multi-racial populations indicates that the average mLDFA is 2.7 degrees valgus, while the mMPTA averages 2.9 degrees varus. However, there is significant variability in these values across different individuals, with a broad range observed for both angles [4]. For the Japanese population, the average values for the mLDFA ranged between 88.5 and 95.0 degrees; for the mMPTA, they varied from 77.2 to 86.4 degrees [5].

The racial differences in the lower limb alignment have been reported in several reports so far. According to a report in 2009, Japanese people have a higher prevalence of varus alignment compared to Caucasians [6]. A study focusing on both tibia and femur variations indicated that the Asian population tends to have greater proximal tibial varus and distal femoral valgus compared to Caucasians [7]. As described above, it was already revealed that there are racial differences in distal femoral and proximal tibial formations among races.

The Coronal Plane Alignment of the Knee (CPAK) classification system, introduced by MacDessi in 2021 [8], provides a structured approach for categorizing the coronal plane alignment of the lower extremities. CPAK classification system segments coronal plane alignment into nine distinct knee types, utilizing measurements from the arithmetic hip-knee-ankle angle (aHKA) and joint line obliquity (JLO). Described as simple and comprehensive, this system effectively delineates coronal plane alignment in arthritic and healthy knees. It provides surgeons with a valuable tool for determining the optimal alignment strategy tailored to each patient’s needs [8].

Research on the CPAK classification system has been conducted in several countries. Among healthy young adults in Belgium and Taiwan, Type II is the most common classification, with Type I being the next most common [8,9]. Regarding total knee arthroplasty (TKA) cases, MacDessi reported that Type II is the most prevalent type in the Australian population [8]. Conversely, Toyooka found that Type I was the most common classification among Japanese cases undergoing TKA [5]. These results indicate the possibility of racial variations in CPAK classifications.

While CPAK classifications have been explored in multiple countries [10,11], few studies have been conducted in Japan [5,12]. Furthermore, there are no existing studies on the CPAK classification among the Japanese population unaffected by knee osteoarthritis (OA), nor on how aging and the progression of OA impact the distribution of CPAK classifications. This study aims to assess the effects of age and the progression of knee OA on the coronal plane alignment of the lower extremity in Japanese patients and to analyze the differences in the distribution of CPAK classification between male and female subjects.

## 2. Materials and Methods

### 2.1. Patients

This study was conducted following the ethical standards of the Declaration of Helsinki and approved by the Institutional Review Board of Tohoku University Hospital (approval number 2022-1-066). This retrospective study included consecutive patients presenting with knee complaints at our clinic between January 2018 and December 2022. These patients were stratified into three groups based on age and the progression of knee OA, as defined by the Kellgren-Lawrence (KL) classification [13]. Group A included patients aged 20–49 years with a KL classification of Grade 0–2; Group B comprised patients aged 50 years or older with KL Grade 0–2; and Group C consisted of patients aged 50 years or older with KL Grade 3–4. Each group contained an equal number of 50 patients per gender. Patients with conditions that could compromise the accuracy of alignment measurements or affect coronal alignment were excluded from the study. The exclusion criteria included: (1) patients with end-stage hip OA; (2) those with end-stage ankle OA; (3) individuals with joint destruction due to rheumatoid arthritis, idiopathic osteonecrosis of the knee, steroid-induced arthropathy, or hemophilic arthritis; (4) patients with knee flexion contracture more than 20 degrees; and (5) those with lower leg implants such as joint prostheses, plates, or intramedullary nails due to around knee osteotomy and trauma.

### 2.2. Radiographic Measurements

All patients underwent standard digital long-leg radiographs. A single orthopedic surgeon at our hospital carried out measurements. The mLDFA and mMPTA were measured, and the aHKA (mMPTA − mLDFA) and the JLO (mMPTA + mLDFA) were calculated. The mLDFA was defined as the lateral angle between the femoral mechanical axis and the distal femoral joint line. The mMPTA was defined as the medial angle between the tibial mechanical axis and the proximal tibia’s joint line. The aHKA is the value of assessing constitutional coronal alignment that excludes the effects of cartilage and bone loss associated with knee OA progression [14]. JLO refers to the inclination of the knee joint line relative to the mechanical axis.

### 2.3. CPAK Classification

**Figure 1:**
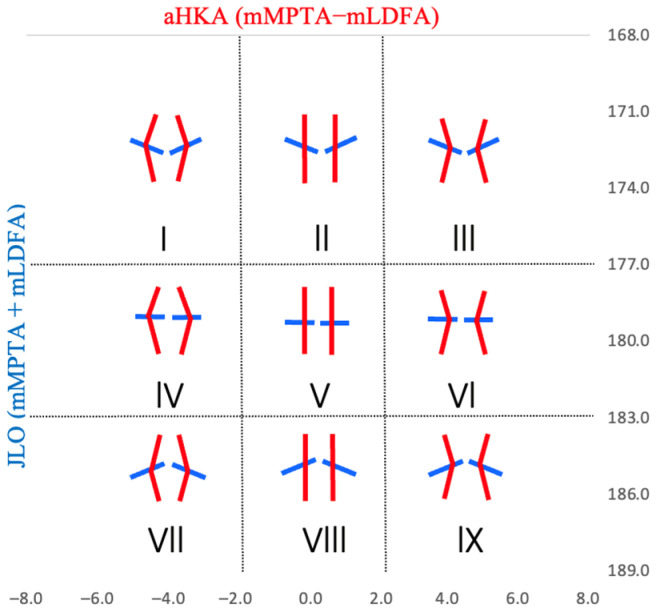
The CPAK classifications.

The classification of this study was based on the original CPAK classification proposed by MacDessi in 2021 [8]. Figure 1 shows the original CPAK classification. The classification in this study was based on the two independent variables of aHKA and JLO, which were divided into three subtypes each, resulting in nine distinct phenotypes. According to the original CPAK classification, the range for neutral aHKA is defined from −2° to 2°. Values below −2° were categorized as varus, while those above 2° were considered valgus. Neutral JLO was defined as ranging between 177° and 183°; values below 177° were classified as apex distal, and values above 183° were classified as apex proximal. CPAK Types I to III, characterized by an apex distal JLO, were differentiated by aHKA values as follows: Type I exhibited a varus aHKA alignment, Type II was neutral, and Type III presented a valgus alignment. Types IV to VI, which feature a neutral JLO, and Types VII to IX, identified by an apex proximal JLO, were similarly categorized into varus, neutral, and valgus alignments according to their aHKA values [8].

### 2.4. Statical Analysis

All data are expressed as the mean ± standard deviation (SD). Data normality was examined using the Shapiro–Wilk test. Statistical differences were assessed using Bonferroni correction, *t*-tests for parametric data, and Wilcoxon test for non-parametric data through JMP 17 data analysis software (SAS Institute Japan, Tokyo, Japan). In multiple comparisons with k null hypotheses, the significance level α was adjusted to α/k by Bonferroni correction, with *p* < 0.05/k deemed statistically significant. Since two comparisons were conducted in this study—between Group A and Group B and between Group B and Group C—the significance level was set at *p* < 0.025, calculated as 0.05/2. Scatter plots were created to show the alignment distribution of CPAK classification.

This figure shows the original CPAK classifications. The classification system segments coronal plane alignment into nine distinct knee types based on aHKA and JLO. The vertical lines in each type indicate the coronal plane alignment by aHKA, and the horizontal lines indicate the inclination of the knee joint inclination by JLO. For reference, mLDFA, mechanical lateral distal femoral angle; mMPTA, mechanical medial proximal tibial angle; aHKA, arithmetic hip-knee-ankle angle; JLO, joint line obliquity.

## 3. Results

### 3.1. Radiographic Measurement

Mean and standard deviation (SD) values for age, mLDFA, mMPTA, aHKA, and JLO are shown in Table 1. The following trends were observed in these values: mLDFA analysis showed a trend toward stronger varus alignment with age and progression of OA in men (Group A vs. Group B, *p* = 0.003; Group B vs. Group C, *p* = 0.001). In women, there was no significant difference with age (Group A vs. Group B, *p* = 0.439), but alignment became more varus as knee OA progressed (Group B vs. Group C, *p* = 0.008). The mMPTA analysis showed no significant difference in men across all groups, suggesting that alignment did not change with age or OA. (Group A vs. Group B, *p* = 0.359; Group B vs. Group C, *p* = 0.124). In women, alignment tended to become more arthritic with age and OA (Group A vs. Group B, *p* = 0.049; Group B vs. Group C, *p* = 0.004). The aHKA analysis showed that in men, only knee osteoarthritis significantly affected varus alignment in all legs (Group A vs. Group B, *p* = 0.263; Group B vs. Group C, *p* = 0.001). In women, both knee osteoarthritis and aging affected overall leg alignment (Group A vs. Group B, *p* = 0.049; Group B vs. Group C, *p* = 0.001). These results indicate that trends in femoral and tibial mass alignment with aging and OA differ between men and women. In contrast, JLO did not show significant variations between groups of either gender, with most patients categorized as apex distal. This suggests that knee joint line inclination remains unaffected by aging and OA, and most patients retain an apex distal classification.

### 3.2. CPAK Classification

**Figure 2:**
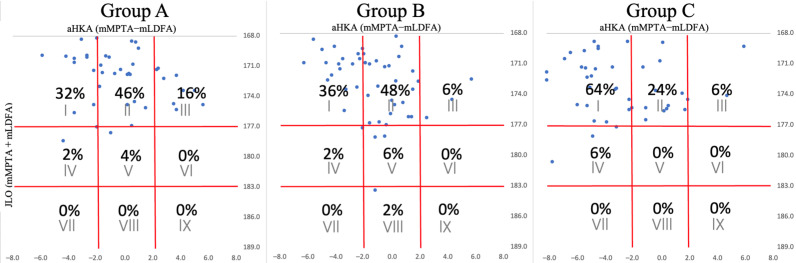
CPAK classifications in males.

**Figure 3:**
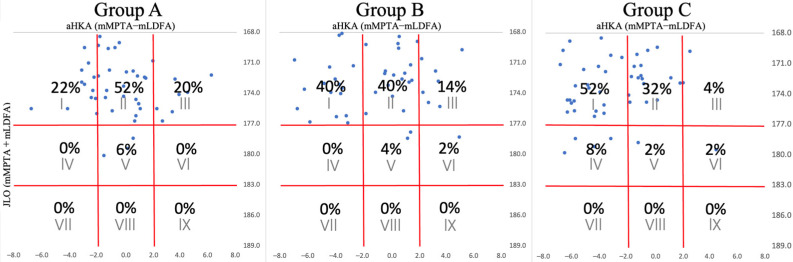
CPAK classifications in females.

Figure 2 and Figure 3 show the CPAK classification distribution and the proportion of each type. For males, Type II predominated in Group A at 46%, followed by Type I at 32%, and Type III at 16%. Group B closely mirrored this pattern, with Type II at 48%, Type I at 36%, and Type III at 6%. However, Group C displayed a significant shift, with Type I becoming the dominant type at 64%, followed by Type II at 24% (Figure 2). This marked change from Groups A and B to Group C signals a shift from Type II to Type I as knee OA advances.

For females, Type II was the most prevalent in Group A at 52%, followed by Type I at 22% and Type III at 20%. In Group B, Types I and II were equally represented at 40%, with Type III at 14%. Group C saw a predominance of Type I at 52%, followed by Type II at 32% and Type IV at 8% (Figure 3). These patterns indicate a transition from Type II to Type I with aging and the progression of knee OA in women, a trend that differs from that observed in men. These data corroborate the findings of the study, highlighting gender-specific differences in the evolution of CPAK classifications with OA progression and aging.

These figures show the percentage distributions for each of the 50 patients in these groups, organized by CPAK classification. The numbers at the bottom of the table represent aHKA values, while those on the right represent JLO values. Plots corresponding to each case are positioned above the CPAK table and are aligned according to aHKA and JLO values. For reference, Group A, patients aged 20–49 years with a KL classification of Grade 0–2; Group B, patients aged 50 years or older with KL Grade 0–2; and Group C, patients aged 50 years or older with KL Grade 3–4.

## 4. Discussion

This study presents the first account of CPAK distribution in the Japanese population without knee OA. The distribution of CPAK in young Japanese people was similar to that in other ethnic groups. However, OA progression in this group was linked to a natural varus alignment in both genders. Interestingly, this alignment’s association with aging was only observed in women, suggesting a potential gender-specific impact on knee alignment changes and OA progression.

This study found that trends in mLDFA and mMPTA varied between males and females. It also revealed that aging impacts aHKA differently by gender, as only OA progression affected males, whereas both OA progression and aging influenced females. These differences might stem from higher osteoporosis rates in females and lifestyle variations, such as the more frequent knee-sitting posture among Japanese women than men [15]. Most patients fell into the JLO apex distal category, and JLO distributions were consistent across different groups and genders. These findings indicate that knee OA leads to increased varus alignment in both genders while preserving JLO.

Age-related changes in CPAK classification in our study revealed distinct patterns between genders. In males, the distribution was relatively similar over time. This similarity is likely due to the aHKA showing no significant changes with age and the JLO consistently staying at apex distal as they aged. On the other hand, a significant change was noted in women with aging; the proportion of Type II decreased while Type I increased. This trend correlates with the aHKA becoming smaller with age.

Considering OA-related changes in CPAK classification, both genders exhibited a notable shift from Type II to Type I as OA progressed. This shift is linked to an increasing varus deformation of the lower leg with OA advancement in both males and females. While the aHKA changed with OA progression, the JLO consistently remained apex in both genders, indicating that both genders tend toward a varus alignment while maintaining medial joint tilt as OA advances.

Comparing the CPAK classification of young people in this study with reports from other countries, we found some similarities and slight differences. In our study of young populations, Type II was the most prevalent CPAK classification, followed by Type I and Type III for both genders. Our findings align with those from Belgium, where Type II (39.2%) was the most common, followed by Type I (26.4%) and Type III (9.8%) [8]. Similarly, in Taiwan, Type II was the most prevalent CPAK classification at 39.3%, followed by Type I at 36.4% and Type III at 13.6% [9]. A comparison of our study with the report from Taiwan showed similarities, with almost 90% of cases classified as JLO-apex distal and similar proportions of each type in Japan and Taiwan. In contrast, among the Chinese population, while Type II was the most prevalent at 44.9%, the second most common type was Type III (23.8%), followed by Type I (22.9%) [16]. These results, along with our report, indicate that Chinese individuals have a higher incidence of valgus knees compared to Japanese individuals, despite both being of the same Asian race. Moreover, in Belgium, about 75% of cases were categorized as JLO-apex distal, and the proportion of Type V (15.7%) is notably higher than that in our study (male: 4%; female: 6%) and Taiwan (4.7%). These results suggest that the European population has a higher incidence of JLO-neutral cases than the Asian population, and there may be regional differences between the same race and different races in terms of the CPAK classification.

Comparing our CPAK classification results for knee OA cases with those from other countries, MacDessi found that in Australia, the most common type was Type II (32.2%), followed by Type I (19.4%) and Type III (15.4%). In contrast, our study revealed that the most common type in the Japanese population was Type I (male: 64%, female: 52%). Similarly, in Chinese people, the most prevalent type was Type I (43.6%) [16]. These findings suggest that racial variations exist in the CPAK classification of knee OA cases, with Japanese knee joints tending to exhibit more varus alignment compared to non-Asian populations.

Several studies have already reported the usefulness of the CPAK classification in surgical planning. In one study focused on unilateral knee arthroplasty, postoperative outcomes were found to be better in the group that retained their preoperative CPAK classification [17]. Numerous comparative studies on TKA have been conducted to determine whether the target alignment should be kinematic or mechanical, and the debate remains ongoing. However, recent reports on treatment outcomes have also incorporated the use of the CPAK classification [18]. In summary, it can be concluded that preoperative identification of a patient’s individual CPAK classification is crucial for accurately categorizing the knee joint and achieving favorable postoperative outcomes.

Our study has emphasized the differences in CPAK classifications between males and females. A report on gender differences in the CPAK classification has been published from Australia, indicating that understanding these gender differences is useful for preoperative planning [10]. The gender differences highlighted in this study suggest that future surgical planning may incorporate gender-specific considerations alongside individualized treatment. Additionally, our study identified morphological changes in the knee joint associated with aging and osteoarthritis. We also observed age-related distribution changes and found significant gender differences in these changes. While age-related distribution changes have been discussed in the original CPAK classification paper [8], no reports have been made in Japan until now. We believe that a comprehensive understanding of the effects of aging will enhance the ability to predict future alterations in knee morphology following surgery. This knowledge will be invaluable for knee surgeons in determining the most appropriate surgical strategies, particularly when addressing conservative treatments for osteoarthritis or undertaking procedures such as ligament and meniscus repair.

This study has several limitations, as described. Firstly, this is a retrospective cross-sectional study. It does not provide a longitudinal evaluation of changes in knee joint alignment in actual patients. In the future, it will be necessary to conduct a prospective longitudinal study. Secondly, the study defines the prevalence of knee OA using a boundary of KL grade 2/3; notably, KL grade 2 is often viewed as early-stage OA, which may influence prevalence interpretation. Thirdly, the imaging assessments were conducted by a single orthopedic surgeon at our institution, which did not allow inter-rater reliability testing and could introduce bias. Future research should include multiple clinicians to ensure the validation and refinement of the findings. Lastly, though the study intended Group A to represent a younger and healthier demographic, these participants were patients visiting our outpatient clinic for various knee issues, indicating that they may not be entirely free from knee health problems. Acknowledging these limitations is essential for interpreting our findings accurately and underscores the need for more methodologically robust and large-scale studies in the future. As this study is cross-sectional, further research is needed to prospectively track changes in CPAK classification and to clarify the impact of aging and the development of osteoarthritis on classification across different racial groups. Such studies are essential for enhancing surgical planning in knee procedures.

## 5. Conclusions

The CPAK classification analysis in the Japanese demographic revealed that OA progression correlates with an inherent varus alignment among both men and women. Aging was notably associated with varus alignment only in women. This difference highlights how gender might affect the interaction between aging and changes in knee alignment during OA progression.

## Figures and Tables

**Table 1:** Comparison of radiographic parameters between each group.

		Group A	Group B	Group C	*p*-Value A vs. B	*p*-Value B vs. C
Male	mLDFA	86.0 ± 1.7	87.1 ± 1.6	88 ± 2.2	0.003	0.001
	mMPTA	85.3 ± 2.4	85.8 ± 2.3	84.5 ± 2.7	0.359	0.124
	aHKA	−0.7 ± 2.8	−1.3 ± 2.4	−3.5 ± 3.7	0.263	0.001
	JLO	171.3 ± 3.1	172.8 ± 3.2	172.5 ± 3.2	0.292	0.584
Female	mLDFA	86.5 ± 2.1	86.9 ± 2.2	88.1 ± 2.2	0.439	0.008
	mMPTA	86.5 ± 1.9	85.7 ± 2.3	85.0 ± 2.3	0.049	0.004
	aHKA	0.0 ± 2.5	−1.2 ± 3.2	−3.6 ± 2.9	0.018	0.001
	JLO	173.1 ± 3.1	172.5 ± 3.0	173.1 ± 3.3	0.408	0.389

Data are shown as mean ± standard deviation; a *p*-value of <0.025 is considered significant by Student’s *t*-test and Bonferroni correction.

## Data Availability

The data supporting the findings of this study are available in the Supplementary Materials. The Supplementary Materials Section includes all relevant datasets generated and analyzed during the current study. These datasets have been organized to ensure the transparency and reproducibility of the results. Further inquiries can be directed to the corresponding author.

## Supplementary Materials

The following supporting information can be downloaded at: https://www.mdpi.com/article/10.3390/jcm13206250/s1, Table S1: Data on knee joint alignment in male patients in this study; Table S2: Data on knee joint alignment in female patients in this study.

## References

[1] Fang D.M., Ritter M.A., Davis K.E. (2009). Coronal alignment in total knee arthroplasty: Just how important is it?. J. Arthroplast..

[2] Yazdanpanah O., Mobarakeh M.K., Nakhaei M., Baneshi M.R. (2017). Comparison of Double and Single Leg Weight-Bearing Radiography in Determining Knee Alignment. Arch. Bone Jt. Surg..

[3] Kim G.W., Kang J.K., Song E.K., Seon J.K. (2021). Increased joint obliquity after open-wedge high tibial osteotomy induces pain in the lateral compartment: A comparative analysis of the minimum 4-year follow-up outcomes using propensity score matching. Knee Surg. Sports Traumatol. Arthrosc..

[4] Almaawi A.M., Hutt J.R.B., Masse V., Lavigne M., Vendittoli P.A. (2017). The Impact of Mechanical and Restricted Kinematic Alignment on Knee Anatomy in Total Knee Arthroplasty. J. Arthroplast..

[5] Toyooka S., Osaki Y., Masuda H., Arai N., Miyamoto W., Ando S., Kawano H., Nakagawa T. (2023). Distribution of Coronal Plane Alignment of the Knee Classification in Patients with Knee Osteoarthritis in Japan. J. Knee Surg..

[6] Hovinga K.R., Lerner A.L. (2009). Anatomic variations between Japanese and Caucasian populations in the healthy young adult knee joint. J. Orthop. Res..

[7] Micicoi G., Jacquet C., Sharma A., LiArno S., Faizan A., Kley K., Parratte S., Ollivier M. (2021). Neutral alignment resulting from tibial vara and opposite femoral valgus is the main morphologic pattern in healthy middle-aged patients: An exploration of a 3D-CT database. Knee Surg. Sports. Traumatol. Arthrosc..

[8] MacDessi S.J., Griffiths-Jones W., Harris I.A., Bellemans J., Chen D.B. (2021). Coronal Plane Alignment of the Knee (CPAK) classification. Bone Jt. J..

[9] Hsu C.E., Chen C.P., Wang S.P., Huang J.T., Tong K.M., Huang K.C. (2022). Validation and modification of the Coronal Plane Alignment of the Knee classification in the Asian population. Bone Jt. Open.

[10] Huber S., Mitterer J.A., Vallant S.M., Simon S., Hanak-Hammerl F., Schwarz G.M., Klasan A., Hofstaetter J.G. (2023). Gender-specific distribution of knee morphology according to CPAK and functional phenotype classification: Analysis of 8739 osteoarthritic knees prior to total knee arthroplasty using artificial intelligence. Knee Surg. Sports Traumatol. Arthrosc..

[11] Pagan C.A., Karasavvidis T., Lebrun D.G., Jang S.J., MacDessi S.J., Vigdorchik J.M. (2023). Geographic Variation in Knee Phenotypes Based on the Coronal Plane Alignment of the Knee Classification: A Systematic Review. J. Arthroplast..

[12] Nomoto K., Hanada M., Hotta K., Matsuyama Y. (2023). Distribution of coronal plane alignment of the knee classification does not change as knee osteoarthritis progresses: A longitudinal study from the Toei study. Knee Surg. Sports Traumatol. Arthrosc..

[13] Kellgren J.H., Lawrence J.S. (1957). Radiological assessment of osteo-arthrosis. Ann. Rheum. Dis..

[14] MacDessi S.J., Griffiths-Jones W., Harris I.A., Bellemans J., Chen D.B. (2020). The arithmetic HKA (aHKA) predicts the constitutional alignment of the arthritic knee compared to the normal contralateral knee: A matched-pairs radiographic study. Bone Jt. Open.

[15] De Martinis M., Sirufo M.M., Polsinelli M., Placidi G., Di Silvestre D., Ginaldi L. (2021). Gender Differences in Osteoporosis: A Single-Center Observational Study. World J. Mens. Health.

[16] Gao Y.H., Qi Y.M., Huang P.H., Zhao X.Y., Qi X. (2024). Distribution of coronal plane alignment of the knee classification in Chinese osteoarthritic and healthy population: A retrospective cross-sectional observational study. Int. J. Surg..

[17] Kim S.E., Yun K.R., Lee J.M., Lee M.C., Han H.S. (2024). Preserving coronal knee alignment of the knee (CPAK) in unicompartmental knee arthroplasty correlates with superior patient-reported outcomes. Knee Surg. Relat. Res..

[18] Arai N., Toyooka S., Masuda H., Kawano H., Nakagawa T. (2024). Kinematic Alignment Achieves a More Balanced Total Knee Arthroplasty than Mechanical Alignment among CPAK Type I Patients: A Simulation Study. J. Clin. Med..

