# Supplementary material for: Impact of Aging and Knee Osteoarthritis on Lower Limb Alignment and CPAK Classification: Gender Differences in a Japanese Cohort
Source: J Clin Med. 2024 Oct 19;13(20):6250. doi: 10.3390/jcm13206250 (PMC11508215; doi:10.3390/jcm13206250)
Supplement: Supplementary file 1 [file jcm-13-06250-s001.zip › jcm-3237949-supplementary.pdf]

Table S1. Data on knee joint alignment in male patients in this study

| Patient number | Age | Sex | Group | mLDFA | mMPTA | mHKA | aHKA | JLO | CPAK aHKA | CPAK 147/258/369 | CPAK JLO | CPAK JLO LEFT | CPAK type |
|---|---|---|---|---|---|---|---|---|---|---|---|---|---|
| M001 | 49 | M | A | 88.6 | 85.0 | −3.6 | −3.6 | 173.6 | varus | 147 | AD | 1 | 1 |
| M002 | 27 | M | A | 87.1 | 83.5 | −2.4 | −3.6 | 170.6 | varus | 147 | AD | 1 | 1 |
| M003 | 24 | M | A | 86.9 | 87.6 | −0.2 | 0.7 | 174.5 | neutral | 258 | AD | 1 | 2 |
| M004 | 48 | M | A | 85.1 | 83.1 | −1.3 | −2.0 | 168.2 | neutral | 258 | AD | 1 | 2 |
| M005 | 45 | M | A | 91.4 | 87.0 | −1.2 | −4.4 | 178.4 | varus | 147 | neutral | 2 | 4 |
| M006 | 45 | M | A | 91.4 | 80.6 | −10.6 | −10.8 | 172.0 | varus | 147 | AD | 1 | 1 |
| M007 | 45 | M | A | 89.6 | 86.0 | −2.5 | −3.6 | 175.6 | varus | 147 | AD | 1 | 1 |
| M008 | 36 | M | A | 86.9 | 79.6 | −7.7 | −7.3 | 166.5 | varus | 147 | AD | 1 | 1 |
| M009 | 37 | M | A | 86.8 | 84.6 | −2.6 | −2.2 | 171.4 | varus | 147 | AD | 1 | 1 |
| M010 | 49 | M | A | 86.6 | 85.0 | −2.4 | −1.6 | 171.6 | neutral | 258 | AD | 1 | 2 |
| M011 | 25 | M | A | 87.1 | 82.9 | −6.4 | −4.2 | 170.0 | varus | 147 | AD | 1 | 1 |
| M012 | 37 | M | A | 84.7 | 83.0 | −4.5 | −1.7 | 167.7 | neutral | 258 | AD | 1 | 2 |
| M013 | 49 | M | A | 86.2 | 85.5 | −1.8 | −0.7 | 171.7 | neutral | 258 | AD | 1 | 2 |
| M014 | 37 | M | A | 85.7 | 82.6 | −5.7 | −3.1 | 168.3 | varus | 147 | AD | 1 | 1 |
| M015 | 44 | M | A | 89.5 | 87.5 | −2.9 | −2.0 | 177.0 | neutral | 258 | neutral | 2 | 5 |
| M016 | 46 | M | A | 85.8 | 86.0 | −0.8 | 0.2 | 171.8 | neutral | 258 | AD | 1 | 2 |
| M017 | 29 | M | A | 85.1 | 85.7 | −0.2 | 0.6 | 170.8 | neutral | 258 | AD | 1 | 2 |
| M018 | 43 | M | A | 84.7 | 87.5 | 0.7 | 2.8 | 172.2 | valgus | 369 | AD | 1 | 3 |
| M019 | 49 | M | A | 84.0 | 83.1 | −1.6 | −0.9 | 167.1 | neutral | 258 | AD | 1 | 2 |
| M020 | 30 | M | A | 85.6 | 89.1 | 4.6 | 3.5 | 174.7 | valgus | 369 | AD | 1 | 3 |
| M021 | 46 | M | A | 86.3 | 83.6 | −4.1 | −2.7 | 169.9 | varus | 147 | AD | 1 | 1 |
| M022 | 15 | M | A | 86.4 | 83.7 | −5.6 | −2.7 | 170.1 | varus | 147 | AD | 1 | 1 |
| M023 | 21 | M | A | 87.9 | 82.0 | −7.6 | −5.9 | 169.9 | varus | 147 | AD | 1 | 1 |
| M024 | 20 | M | A | 85.8 | 85.5 | −0.2 | −0.3 | 171.3 | neutral | 258 | AD | 1 | 2 |
| M025 | 35 | M | A | 85.8 | 89.5 | 4.6 | 3.7 | 175.3 | valgus | 369 | AD | 1 | 3 |

| | | | | | | | | | | | | | |
|---|---|---|---|---|---|---|---|---|---|---|---|---|---|
| M026 | 46 | M | A | 84.1 | 85.1 | 1.4 | 1.0 | 169.2 | neutral | 258 | AD | 1 | 2 |
| M027 | 44 | M | A | 83.4 | 84.0 | 0.2 | 0.6 | 167.4 | neutral | 258 | AD | 1 | 2 |
| M028 | 39 | M | A | 89.3 | 88.3 | −4.0 | −1.0 | 177.6 | neutral | 258 | neutral | 2 | 5 |
| M029 | 20 | M | A | 84.2 | 89.2 | 5.0 | 5.0 | 173.4 | valgus | 369 | AD | 1 | 3 |
| M030 | 23 | M | A | 84.0 | 84.5 | 0.7 | 0.5 | 168.5 | neutral | 258 | AD | 1 | 2 |
| M031 | 41 | M | A | 84.6 | 88.8 | 3.5 | 4.2 | 173.4 | valgus | 369 | AD | 1 | 3 |
| M032 | 47 | M | A | 86.8 | 88.3 | 2.8 | 1.5 | 175.1 | neutral | 258 | AD | 1 | 2 |
| M033 | 43 | M | A | 85.7 | 84.3 | −1.6 | −1.36 | 170.0 | neutral | 258 | AD | 1 | 2 |
| M034 | 29 | M | A | 85.7 | 86.1 | −1.8 | 0.4 | 171.8 | neutral | 258 | AD | 1 | 2 |
| M035 | 42 | M | A | 85.7 | 86.0 | −2.1 | 0.3 | 171.7 | neutral | 258 | AD | 1 | 2 |
| M036 | 35 | M | A | 84.6 | 90.2 | 3.1 | 5.6 | 174.8 | valgus | 369 | AD | 1 | 3 |
| M037 | 38 | M | A | 87.4 | 85.6 | −4.3 | −1.8 | 173.0 | neutral | 258 | AD | 1 | 2 |
| M038 | 48 | M | A | 83.8 | 83.9 | 0.2 | 0.1 | 167.7 | neutral | 258 | AD | 1 | 2 |
| M039 | 22 | M | A | 86.5 | 80.9 | −3.5 | −5.6 | 167.4 | varus | 147 | AD | 1 | 1 |
| M040 | 20 | M | A | 84.1 | 87.8 | −0.3 | 3.7 | 171.9 | valgus | 369 | AD | 1 | 3 |
| M041 | 29 | M | A | 88.2 | 88.7 | −2.8 | 0.5 | 176.9 | neutral | 258 | AD | 1 | 2 |
| M042 | 36 | M | A | 84.8 | 82.2 | −2.4 | −2.6 | 167.0 | varus | 147 | AD | 1 | 1 |
| M043 | 33 | M | A | 84.8 | 83.0 | −2.3 | −1.8 | 167.8 | neutral | 258 | AD | 1 | 2 |
| M044 | 42 | M | A | 84.4 | 86.8 | 0.4 | 2.4 | 171.2 | valgus | 369 | AD | 1 | 3 |
| M045 | 31 | M | A | 88.5 | 86.3 | −4.4 | −2.2 | 174.8 | varus | 147 | AD | 1 | 1 |
| M046 | 33 | M | A | 85.6 | 84.5 | −1.3 | −1.1 | 170.1 | neutral | 258 | AD | 1 | 2 |
| M047 | 38 | M | A | 83.9 | 84.7 | −0.3 | 0.8 | 168.6 | neutral | 258 | AD | 1 | 2 |
| M048 | 33 | M | A | 86.3 | 84.6 | −0.9 | −1.7 | 170.9 | neutral | 258 | AD | 1 | 2 |
| M049 | 42 | M | A | 86.9 | 83.3 | −2.8 | −3.6 | 170.2 | varus | 147 | AD | 1 | 1 |
| M050 | 38 | M | A | 85.4 | 80.6 | −6.2 | −4.8 | 166.0 | varus | 147 | AD | 1 | 1 |
| M051 | 85 | M | B | 87.4 | 88.8 | 0.6 | 1.4 | 176.2 | neutral | 258 | AD | 1 | 2 |
| M052 | 61 | M | B | 86.3 | 84.7 | −1.8 | −1.6 | 171.0 | neutral | 258 | AD | 1 | 2 |
| M053 | 69 | M | B | 87.4 | 83.6 | −3.9 | −3.8 | 171.0 | varus | 147 | AD | 1 | 1 |
| M054 | 51 | M | B | 86.9 | 84.1 | −1.4 | −2.8 | 171.0 | varus | 147 | AD | 1 | 1 |
| M055 | 61 | M | B | 86.0 | 84.0 | −2.6 | −2.0 | 170.0 | neutral | 258 | AD | 1 | 2 |

| | | | | | | | | | | | | | |
|---|---|---|---|---|---|---|---|---|---|---|---|---|---|
| M056 | 61 | M | B | 85.9 | 85.4 | −1.3 | −0.5 | 171.3 | neutral | 258 | AD | 1 | 2 |
| M057 | 76 | M | B | 86.2 | 86.5 | 0.6 | 0.3 | 172.7 | neutral | 258 | AD | 1 | 2 |
| M058 | 60 | M | B | 85.1 | 80.7 | −9.5 | −4.4 | 165.8 | varus | 147 | AD | 1 | 1 |
| M059 | 77 | M | B | 86.4 | 84.5 | −3.2 | −1.9 | 170.9 | neutral | 258 | AD | 1 | 2 |
| M060 | 50 | M | B | 88.2 | 83.9 | −2.9 | −4.3 | 172.1 | varus | 147 | AD | 1 | 1 |
| M061 | 61 | M | B | 84.0 | 84.3 | 0.1 | 0.3 | 168.3 | neutral | 258 | AD | 1 | 2 |
| M062 | 78 | M | B | 87.9 | 86.2 | −0.9 | −1.7 | 174.1 | neutral | 258 | AD | 1 | 2 |
| M063 | 61 | M | B | 86.7 | 82.6 | −5.4 | −4.1 | 169.3 | varus | 147 | AD | 1 | 1 |
| M064 | 84 | M | B | 88.7 | 88.3 | −1.9 | −0.4 | 177.0 | neutral | 258 | neutral | 2 | 5 |
| M065 | 25 | M | B | 88.6 | 82.3 | −6.5 | −6.3 | 170.9 | varus | 147 | AD | 1 | 1 |
| M066 | 85 | M | B | 89.2 | 87.6 | −1.7 | −1.6 | 176.8 | neutral | 258 | AD | 1 | 2 |
| M067 | 82 | M | B | 87.3 | 82.3 | −4.5 | −5.0 | 169.6 | varus | 147 | AD | 1 | 1 |
| M068 | 66 | M | B | 87.8 | 83.1 | −7.2 | −4.7 | 170.9 | varus | 147 | AD | 1 | 1 |
| M069 | 60 | M | B | 86.4 | 84.5 | −1.8 | −1.9 | 170.9 | neutral | 258 | AD | 1 | 2 |
| M070 | 77 | M | B | 89.5 | 86.1 | −4.3 | −3.4 | 175.6 | varus | 147 | AD | 1 | 1 |
| M071 | 57 | M | B | 92.3 | 91.1 | −1.0 | −1.2 | 183.4 | neutral | 258 | AP | 3 | 8 |
| M072 | 55 | M | B | 87.0 | 83.5 | −3.3 | −3.5 | 170.5 | varus | 147 | AD | 1 | 1 |
| M073 | 55 | M | B | 83.4 | 89.1 | 2.8 | 5.7 | 172.5 | valgus | 369 | AD | 1 | 3 |
| M074 | 59 | M | B | 89.7 | 87.6 | −4.9 | −2.1 | 177.3 | varus | 147 | neutral | 2 | 4 |
| M075 | 56 | M | B | 85.8 | 83.7 | −1.7 | −2.1 | 169.5 | varus | 147 | AD | 1 | 1 |
| M076 | 53 | M | B | 86.2 | 83.8 | −2.7 | −2.4 | 170.0 | varus | 147 | AD | 1 | 1 |
| M077 | 63 | M | B | 86.0 | 87.5 | 0.7 | 1.5 | 173.5 | neutral | 258 | AD | 1 | 2 |
| M078 | 57 | M | B | 86.4 | 84.1 | −0.5 | −2.3 | 170.5 | varus | 147 | AD | 1 | 1 |
| M079 | 73 | M | B | 88.8 | 87.9 | −3.6 | −0.9 | 176.7 | neutral | 258 | AD | 1 | 2 |
| M080 | 53 | M | B | 87.1 | 86.1 | −6.8 | −1.0 | 173.2 | neutral | 258 | AD | 1 | 2 |
| M081 | 59 | M | B | 88.4 | 85.1 | −4.9 | −3.3 | 173.5 | varus | 147 | AD | 1 | 1 |
| M082 | 53 | M | B | 87.0 | 87.8 | −2.5 | 0.8 | 174.8 | neutral | 258 | AD | 1 | 2 |
| M083 | 53 | M | B | 85.1 | 86.6 | 1.0 | 1.5 | 171.7 | neutral | 258 | AD | 1 | 2 |
| M084 | 66 | M | B | 88.6 | 84.0 | −5.8 | −4.6 | 172.6 | varus | 147 | AD | 1 | 1 |
| M085 | 62 | M | B | 89.7 | 88.5 | −2.1 | −1.2 | 178.2 | neutral | 258 | neutral | 2 | 5 |

| | | | | | | | | | | | | | |
|---|---|---|---|---|---|---|---|---|---|---|---|---|---|
| M086 | 63 | M | B | 87.3 | 87.3 | −2.0 | 0 | 174.6 | neutral | 258 | AD | 1 | 2 |
| M087 | 71 | M | B | 87.1 | 81.5 | −5.9 | −5.6 | 168.6 | varus | 147 | AD | 1 | 1 |
| M088 | 64 | M | B | 85.7 | 85.5 | −0.9 | −0.2 | 171.2 | neutral | 258 | AD | 1 | 2 |
| M089 | 81 | M | B | 89.3 | 88.8 | −1.2 | −0.5 | 178.1 | neutral | 258 | neutral | 2 | 5 |
| M090 | 87 | M | B | 86.9 | 89.4 | −1.0 | 2.5 | 176.3 | valgus | 369 | AD | 1 | 3 |
| M091 | 50 | M | B | 87.9 | 85.4 | −2.7 | −2.5 | 173.3 | varus | 147 | AD | 1 | 1 |
| M092 | 63 | M | B | 87.6 | 83.9 | −2.9 | −3.7 | 171.5 | varus | 147 | AD | 1 | 1 |
| M093 | 55 | M | B | 84.3 | 85.0 | −1.5 | 0.7 | 169.3 | neutral | 258 | AD | 1 | 2 |
| M094 | 62 | M | B | 85.4 | 87.3 | 2.1 | 1.9 | 172.7 | neutral | 258 | AD | 1 | 2 |
| M095 | 66 | M | B | 88.0 | 87.9 | −1.5 | −0.1 | 175.9 | neutral | 258 | AD | 1 | 2 |
| M096 | 51 | M | B | 84.9 | 85.4 | 0.1 | 0.5 | 170.3 | neutral | 258 | AD | 1 | 2 |
| M097 | 56 | M | B | 85.1 | 89.4 | 1.8 | 4.3 | 174.5 | valgus | 369 | AD | 1 | 3 |
| M098 | 59 | M | B | 85.9 | 84.8 | −0.4 | −1.1 | 170.7 | neutral | 258 | AD | 1 | 2 |
| M099 | 77 | M | B | 87.4 | 87.6 | −2.1 | 0.2 | 175.0 | neutral | 258 | AD | 1 | 2 |
| M100 | 53 | M | B | 87.5 | 88.7 | −1.0 | 1.2 | 176.2 | neutral | 258 | AD | 1 | 2 |
| M101 | 70 | M | C | 90.4 | 84.6 | −9.2 | −5.8 | 175.0 | varus | 147 | AD | 1 | 1 |
| M102 | 63 | M | C | 87.6 | 88.0 | −7.5 | 0.4 | 175.6 | neutral | 258 | AD | 1 | 2 |
| M103 | 70 | M | C | 89.1 | 84.2 | −12.9 | −4.9 | 173.3 | varus | 147 | AD | 1 | 1 |
| M104 | 59 | M | C | 86.1 | 80.0 | −9.2 | −6.1 | 166.1 | varus | 147 | AD | 1 | 1 |
| M105 | 54 | M | C | 87.9 | 78.1 | −12.5 | −9.8 | 166.0 | varus | 147 | AD | 1 | 1 |
| M106 | 73 | M | C | 85.1 | 82.6 | −6.7 | −2.5 | 167.7 | varus | 147 | AD | 1 | 1 |
| M107 | 61 | M | C | 88.4 | 82.9 | −8.0 | −5.5 | 171.3 | varus | 147 | AD | 1 | 1 |
| M108 | 86 | M | C | 88.0 | 83.5 | −4.9 | −4.5 | 171.5 | varus | 147 | AD | 1 | 1 |
| M109 | 73 | M | C | 86.9 | 82.6 | −3.7 | −4.3 | 169.5 | varus | 147 | AD | 1 | 1 |
| M110 | 83 | M | C | 85.4 | 82.1 | −3.9 | −3.3 | 167.5 | varus | 147 | AD | 1 | 1 |
| M111 | 88 | M | C | 87.3 | 84.0 | −4.7 | −3.3 | 171.3 | varus | 147 | AD | 1 | 1 |
| M112 | 72 | M | C | 86.1 | 87.8 | −9.4 | 1.7 | 173.9 | neutral | 258 | AD | 1 | 2 |
| M113 | 76 | M | C | 88.2 | 85.2 | −3.0 | −3.0 | 173.4 | varus | 147 | AD | 1 | 1 |
| M114 | 74 | M | C | 86.8 | 82.5 | 14.7 | −4.3 | 169.3 | varus | 147 | AD | 1 | 1 |
| M115 | 71 | M | C | 88.1 | 87.2 | −8.8 | −0.9 | 175.3 | neutral | 258 | AD | 1 | 2 |

| | | | | | | | | | | | | | |
|---|---|---|---|---|---|---|---|---|---|---|---|---|---|
| M116 | 73 | M | C | 87.2 | 87.9 | −2.4 | 0.7 | 175.1 | neutral | 258 | AD | 1 | 2 |
| M117 | 68 | M | C | 81.6 | 87.7 | 8.4 | 6.1 | 169.3 | valgus | 369 | AD | 1 | 3 |
| M118 | 77 | M | C | 90.3 | 82.3 | −13.3 | −8.0 | 172.6 | varus | 147 | AD | 1 | 1 |
| M119 | 68 | M | C | 86.9 | 86.7 | −3.9 | −0.2 | 173.6 | neutral | 258 | AD | 1 | 2 |
| M120 | 70 | M | C | 91.2 | 82.5 | −10.4 | −8.7 | 173.7 | varus | 147 | AD | 1 | 1 |
| M121 | 68 | M | C | 94.1 | 86.5 | 4.5 | −7.6 | 180.6 | varus | 147 | neutral | 2 | 4 |
| M122 | 72 | M | C | 90.1 | 87.1 | −8.8 | −3.0 | 177.2 | varus | 147 | neutral | 2 | 4 |
| M123 | 79 | M | C | 89.7 | 80.5 | −19.7 | −9.2 | 170.2 | varus | 147 | AD | 1 | 1 |
| M124 | 55 | M | C | 86.2 | 88.3 | 4.6 | 2.1 | 174.5 | valgus | 369 | AD | 1 | 3 |
| M125 | 80 | M | C | 90.1 | 85.0 | −14.7 | −5.1 | 175.1 | varus | 147 | AD | 1 | 1 |
| M126 | 59 | M | C | 86.8 | 88.7 | −5.5 | 1.9 | 175.5 | neutral | 258 | AD | 1 | 2 |
| M127 | 81 | M | C | 85.3 | 85.4 | 0.3 | 0.1 | 170.7 | neutral | 258 | AD | 1 | 2 |
| M128 | 68 | M | C | 88.3 | 83.1 | −11.0 | −5.2 | 171.4 | varus | 147 | AD | 1 | 1 |
| M129 | 56 | M | C | 88.3 | 85.2 | −6.1 | −3.1 | 173.5 | varus | 147 | AD | 1 | 1 |
| M130 | 78 | M | C | 89.9 | 81.9 | −16.3 | −8.0 | 171.8 | varus | 147 | AD | 1 | 1 |
| M131 | 60 | M | C | 90.6 | 86.0 | −8.1 | −4.6 | 176.6 | varus | 147 | AD | 1 | 1 |
| M132 | 72 | M | C | 87.0 | 80.6 | −21.8 | −6.4 | 167.6 | varus | 147 | AD | 1 | 1 |
| M133 | 81 | M | C | 88.8 | 86.7 | −12.3 | −2.1 | 175.5 | varus | 147 | AD | 1 | 1 |
| M134 | 59 | M | C | 88.6 | 81.9 | −12.7 | −6.7 | 170.5 | varus | 147 | AD | 1 | 1 |
| M135 | 60 | M | C | 84.3 | 84.6 | 0.3 | 0.3 | 168.9 | neutral | 258 | AD | 1 | 2 |
| M136 | 76 | M | C | 88.6 | 86.7 | −4.7 | −1.9 | 175.3 | neutral | 258 | AD | 1 | 2 |
| M137 | 78 | M | C | 85.5 | 83.3 | −4.3 | −2.2 | 168.8 | varus | 147 | AD | 1 | 1 |
| M138 | 85 | M | C | 88.7 | 83.6 | −9.7 | −5.1 | 172.3 | varus | 147 | AD | 1 | 1 |
| M139 | 71 | M | C | 88.1 | 81.8 | −6.5 | −6.3 | 169.9 | varus | 147 | AD | 1 | 1 |
| M140 | 67 | M | C | 87.5 | 82.2 | −13.1 | −5.3 | 169.7 | varus | 147 | AD | 1 | 1 |
| M141 | 87 | M | C | 86.2 | 85.1 | −4.3 | −1.1 | 171.3 | neutral | 258 | AD | 1 | 2 |
| M142 | 63 | M | C | 88.7 | 87.8 | −6.1 | −0.9 | 176.5 | neutral | 258 | AD | 1 | 2 |
| M143 | 72 | M | C | 88.5 | 83.7 | −8.1 | −4.8 | 172.2 | varus | 147 | AD | 1 | 1 |
| M144 | 62 | M | C | 88.3 | 86.2 | −3.9 | −2.1 | 174.5 | varus | 147 | AD | 1 | 1 |
| M145 | 80 | M | C | 90.2 | 86.5 | −5.9 | −3.7 | 176.7 | varus | 147 | AD | 1 | 1 |

| | | | | | | | | | | | | | |
|---|---|---|---|---|---|---|---|---|---|---|---|---|---|
| M146 | 73 | M | C | 91.4 | 86.7 | −11.0 | −4.7 | 178.1 | varus | 147 | neutral | 2 | 4 |
| M147 | 55 | M | C | 84.6 | 89.5 | 3.9 | 4.9 | 174.1 | valgus | 369 | AD | 1 | 3 |
| M148 | 71 | M | C | 91.6 | 77.8 | −15.9 | −13.8 | 169.4 | varus | 147 | AD | 1 | 1 |
| M149 | 58 | M | C | 86.7 | 82.1 | −6.5 | −4.6 | 168.8 | varus | 147 | AD | 1 | 1 |
| M150 | 60 | M | C | 87.4 | 88.0 | −2.8 | 0.6 | 175.4 | neutral | 258 | AD | 1 | 2 |

Table S2. Data on knee joint alignment in female patients in this study.

| Patient number | Age | Sex | Group | mLDFA | mMPTA | mHKA | aHKA | JLO | CPAK aHKA | CPAK 147/258/369 | CPAK JLO | CPAK JLO LEFT | CPAK type |
|---|---|---|---|---|---|---|---|---|---|---|---|---|---|
| F001 | 32 | F | A | 88.9 | 89.5 | 0.5 | 0.6 | 178.4 | neutral | 258 | neutral | 2 | 5 |
| F002 | 26 | F | A | 87.9 | 86.5 | 0.1 | −1.4 | 174.4 | neutral | 258 | AD | 1 | 2 |
| F003 | 49 | F | A | 82.9 | 84.3 | 3.9 | 1.4 | 167.2 | neutral | 258 | AD | 1 | 2 |
| F004 | 40 | F | A | 86.8 | 84.2 | −1.1 | −2.6 | 171.0 | varus | 147 | AD | 1 | 1 |
| F005 | 34 | F | A | 86.2 | 89.6 | 1.1 | 3.4 | 175.8 | valgus | 369 | AD | 1 | 3 |
| F006 | 39 | F | A | 85.4 | 84.2 | 1.7 | −1.2 | 169.6 | neutral | 258 | AD | 1 | 2 |
| F007 | 42 | F | A | 85.9 | 86.0 | 0.0 | 0.1 | 171.9 | neutral | 258 | AD | 1 | 2 |
| F008 | 20 | F | A | 82.1 | 84.7 | −0.4 | 2.6 | 166.8 | valgus | 369 | AD | 1 | 3 |
| F009 | 35 | F | A | 88.0 | 84.9 | −2.4 | −3.1 | 172.9 | varus | 147 | AD | 1 | 1 |
| F010 | 22 | F | A | 88.0 | 88.7 | 2.2 | 0.7 | 176.7 | neutral | 258 | AD | 1 | 2 |
| F011 | 23 | F | A | 86.6 | 88.9 | 1.0 | 2.3 | 175.5 | valgus | 369 | AD | 1 | 3 |
| F012 | 45 | F | A | 85.5 | 87.0 | −0.2 | 1.5 | 172.5 | neutral | 258 | AD | 1 | 2 |
| F013 | 26 | F | A | 85.5 | 86.9 | 1.1 | 1.4 | 172.4 | neutral | 258 | AD | 1 | 2 |
| F014 | 37 | F | A | 86.2 | 83.3 | −4.1 | −2.9 | 169.5 | varus | 147 | AD | 1 | 1 |
| F015 | 45 | F | A | 86.4 | 85.3 | −0.9 | −1.1 | 171.7 | neutral | 258 | AD | 1 | 2 |
| F016 | 22 | F | A | 87.4 | 84.3 | −2.3 | −3.1 | 171.7 | varus | 147 | AD | 1 | 1 |
| F017 | 48 | F | A | 87.0 | 88.0 | −0.6 | 1.0 | 175.0 | neutral | 258 | AD | 1 | 2 |
| F018 | 36 | F | A | 85.6 | 86.2 | 0.0 | 0.6 | 171.8 | neutral | 258 | AD | 1 | 2 |
| F019 | 30 | F | A | 87.7 | 88.5 | 0.9 | 0.8 | 176.2 | neutral | 258 | AD | 1 | 2 |
| F020 | 33 | F | A | 84.5 | 88.1 | 1.2 | 3.6 | 172.6 | valgus | 369 | AD | 1 | 3 |

| | | | | | | | | | | | | | |
|---|---|---|---|---|---|---|---|---|---|---|---|---|---|
| F021 | 45 | F | A | 86.7 | 86.6 | −0.4 | −0.1 | 173.3 | neutral | 258 | AD | 1 | 2 |
| F022 | 35 | F | A | 83.0 | 89.2 | 4.1 | 6.2 | 172.2 | valgus | 369 | AD | 1 | 3 |
| F023 | 43 | F | A | 85.6 | 83.7 | −3.2 | −1.9 | 169.3 | neutral | 258 | AD | 1 | 2 |
| F024 | 48 | F | A | 88.3 | 87.4 | −1.0 | −0.9 | 175.7 | neutral | 258 | AD | 1 | 2 |
| F025 | 38 | F | A | 86.9 | 87.7 | 0.1 | 0.8 | 174.6 | neutral | 258 | AD | 1 | 2 |
| F026 | 48 | F | A | 88.4 | 86.0 | −1.5 | −2.4 | 174.4 | varus | 147 | AD | 1 | 1 |
| F027 | 28 | F | A | 89.8 | 85.7 | −2.5 | −4.1 | 175.5 | varus | 147 | AD | 1 | 1 |
| F028 | 48 | F | A | 84.4 | 86.4 | 2.7 | 2.0 | 170.8 | neutral | 258 | AD | 1 | 2 |
| F029 | 46 | F | A | 91.1 | 84.4 | −2.2 | −6.7 | 175.5 | varus | 147 | AD | 1 | 1 |
| F030 | 48 | F | A | 81.6 | 85.5 | 3.2 | 3.9 | 167.1 | valgus | 369 | AD | 1 | 3 |
| F031 | 38 | F | A | 84.7 | 84.3 | −1.0 | −0.4 | 169.0 | neutral | 258 | AD | 1 | 2 |
| F032 | 49 | F | A | 87.2 | 87.1 | −2.5 | −0.1 | 174.3 | neutral | 258 | AD | 1 | 2 |
| F033 | 36 | F | A | 84.7 | 89.2 | 4.3 | 4.5 | 173.9 | valgus | 369 | AD | 1 | 3 |
| F034 | 48 | F | A | 88.3 | 86.2 | −2.0 | −2.1 | 174.5 | varus | 147 | AD | 1 | 1 |
| F035 | 30 | F | A | 89.6 | 89.8 | 0.6 | 0.2 | 179.4 | neutral | 258 | neutral | 2 | 5 |
| F036 | 35 | F | A | 89.0 | 87.0 | −1.4 | −2 | 176.0 | neutral | 258 | AD | 1 | 2 |
| F037 | 28 | F | A | 87.1 | 85.2 | −2.8 | −1.9 | 172.3 | neutral | 258 | AD | 1 | 2 |
| F038 | 46 | F | A | 85.1 | 83.3 | −0.3 | −1.8 | 168.4 | neutral | 258 | AD | 1 | 2 |
| F039 | 38 | F | A | 87.0 | 88.0 | 0.0 | 1 | 175.0 | neutral | 258 | AD | 1 | 2 |
| F040 | 43 | F | A | 88.0 | 85.1 | −1.1 | −2.9 | 173.1 | varus | 147 | AD | 1 | 1 |
| F041 | 33 | F | A | 87.2 | 88.3 | 1.5 | 1.1 | 175.5 | neutral | 258 | AD | 1 | 2 |
| F042 | 32 | F | A | 88.0 | 85.7 | −4.8 | −2.3 | 173.7 | varus | 147 | AD | 1 | 1 |
| F043 | 28 | F | A | 87.0 | 89.7 | 3.6 | 2.7 | 176.7 | valgus | 369 | AD | 1 | 3 |
| F044 | 48 | F | A | 87.5 | 86.2 | −2.5 | −1.3 | 173.7 | neutral | 258 | AD | 1 | 2 |
| F045 | 21 | F | A | 85.1 | 89.0 | 2.7 | 3.89 | 174.1 | valgus | 369 | AD | 1 | 3 |
| F046 | 28 | F | A | 90.8 | 89.3 | −3.4 | −1.5 | 180.1 | neutral | 258 | neutral | 2 | 5 |
| F047 | 29 | F | A | 81.8 | 85.1 | 3.1 | 3.3 | 166.9 | valgus | 369 | AD | 1 | 3 |
| F048 | 30 | F | A | 87.3 | 85.1 | −4.0 | −2.2 | 172.4 | varus | 147 | AD | 1 | 1 |
| F049 | 25 | F | A | 85.7 | 86.6 | 1.9 | 0.9 | 172.3 | neutral | 258 | AD | 1 | 2 |
| F050 | 36 | F | A | 85.1 | 84.4 | −0.9 | −0.7 | 169.5 | neutral | 258 | AD | 1 | 2 |

| | | | | | | | | | | | | | |
|---|---|---|---|---|---|---|---|---|---|---|---|---|---|
| F051 | 57 | F | B | 84.9 | 86.3 | 1.8 | 1.4 | 171.2 | neutral | 258 | AD | 1 | 2 |
| F052 | 75 | F | B | 90.0 | 86.9 | 4.3 | −3.1 | 176.9 | varus | 147 | AD | 1 | 1 |
| F053 | 71 | F | B | 86.1 | 85.7 | 4.0 | −0.4 | 171.8 | neutral | 258 | AD | 1 | 2 |
| F054 | 76 | F | B | 86.7 | 91.6 | 5.6 | 4.9 | 178.3 | valgus | 369 | neutral | 2 | 6 |
| F055 | 63 | F | B | 88.9 | 87.1 | −1.3 | −1.8 | 176.0 | neutral | 258 | AD | 1 | 2 |
| F056 | 51 | F | B | 88.3 | 84.3 | 0.7 | −4.0 | 172.6 | varus | 147 | AD | 1 | 1 |
| F057 | 72 | F | B | 83.5 | 85.4 | 1.3 | 1.9 | 168.9 | neutral | 258 | AD | 1 | 2 |
| F058 | 80 | F | B | 87.1 | 87.2 | −0.9 | 0.1 | 174.3 | neutral | 258 | AD | 1 | 2 |
| F059 | 59 | F | B | 83.7 | 83.3 | −1.6 | −0.4 | 167.0 | neutral | 258 | AD | 1 | 2 |
| F060 | 70 | F | B | 86.0 | 82.3 | −5.1 | −3.7 | 168.3 | varus | 147 | AD | 1 | 1 |
| F061 | 64 | F | B | 89.7 | 86.5 | 0.0 | −3.2 | 176.2 | varus | 147 | AD | 1 | 1 |
| F062 | 80 | F | B | 87.5 | 86.4 | −5.7 | −1.1 | 173.9 | neutral | 258 | AD | 1 | 2 |
| F063 | 58 | F | B | 88.8 | 81.9 | −5.9 | −6.9 | 170.7 | varus | 147 | AD | 1 | 1 |
| F064 | 51 | F | B | 87.9 | 79.5 | −4.4 | −8.4 | 167.4 | varus | 147 | AD | 1 | 1 |
| F065 | 65 | F | B | 91.1 | 84.4 | −7.4 | −6.7 | 175.5 | varus | 147 | AD | 1 | 1 |
| F066 | 79 | F | B | 87.9 | 83.5 | −5.6 | −4.4 | 171.4 | varus | 147 | AD | 1 | 1 |
| F067 | 57 | F | B | 85.8 | 82.3 | −5.9 | −3.5 | 168.1 | varus | 147 | AD | 1 | 1 |
| F068 | 76 | F | B | 88.2 | 89.6 | −1.7 | 1.4 | 177.8 | neutral | 258 | neutral | 2 | 5 |
| F069 | 54 | F | B | 88.4 | 84.7 | −2.5 | −3.7 | 173.1 | varus | 147 | AD | 1 | 1 |
| F070 | 62 | F | B | 90.9 | 82.5 | −7.2 | −8.4 | 173.4 | varus | 147 | AD | 1 | 1 |
| F071 | 62 | F | B | 85.9 | 89.4 | −1.0 | 3.5 | 175.3 | valgus | 369 | AD | 1 | 3 |
| F072 | 81 | F | B | 88.9 | 84.1 | −4.6 | −4.8 | 173.0 | varus | 147 | AD | 1 | 1 |
| F073 | 50 | F | B | 87.4 | 88.3 | −1.4 | 0.9 | 175.7 | neutral | 258 | AD | 1 | 2 |
| F074 | 67 | F | B | 84.0 | 83.5 | 0.2 | −0.5 | 167.5 | neutral | 258 | AD | 1 | 2 |
| F075 | 55 | F | B | 88.8 | 85.2 | −4.5 | −3.6 | 174.0 | varus | 147 | AD | 1 | 1 |
| F076 | 55 | F | B | 85.8 | 88.1 | 2.3 | 2.3 | 173.9 | valgus | 369 | AD | 1 | 3 |
| F077 | 59 | F | B | 85.9 | 86.7 | 1.4 | 0.8 | 172.6 | neutral | 258 | AD | 1 | 2 |
| F078 | 57 | F | B | 87.0 | 84.3 | −0.1 | −2.7 | 171.3 | varus | 147 | AD | 1 | 1 |
| F079 | 69 | F | B | 89.1 | 84.8 | −7.6 | −4.3 | 173.9 | varus | 147 | AD | 1 | 1 |
| F080 | 71 | F | B | 85.6 | 87.1 | −2.2 | 1.5 | 172.7 | neutral | 258 | AD | 1 | 2 |

| | | | | | | | | | | | | | |
|---|---|---|---|---|---|---|---|---|---|---|---|---|---|
| F081 | 71 | F | B | 85.8 | 87.1 | −0.9 | 1.3 | 172.9 | neutral | 258 | AD | 1 | 2 |
| F082 | 56 | F | B | 86.1 | 88.8 | −2.5 | 2.7 | 174.9 | valgus | 369 | AD | 1 | 3 |
| F083 | 59 | F | B | 84.3 | 84.8 | 0.1 | 0.5 | 169.1 | neutral | 258 | AD | 1 | 2 |
| F084 | 57 | F | B | 88.6 | 89.8 | 1.3 | 1.2 | 178.4 | neutral | 258 | neutral | 2 | 5 |
| F085 | 51 | F | B | 83.9 | 84.5 | 0.6 | 0.6 | 168.4 | neutral | 258 | AD | 1 | 2 |
| F086 | 47 | F | B | 85.5 | 83.7 | 0.3 | −1.8 | 169.2 | neutral | 258 | AD | 1 | 2 |
| F087 | 64 | F | B | 84.3 | 87.5 | 4.1 | 3.2 | 171.8 | valgus | 369 | AD | 1 | 3 |
| F088 | 65 | F | B | 88.5 | 85.0 | −5.6 | −3.5 | 173.5 | varus | 147 | AD | 1 | 1 |
| F089 | 75 | F | B | 90.1 | 86.2 | −2.4 | −3.9 | 176.3 | varus | 147 | AD | 1 | 1 |
| F090 | 67 | F | B | 85.0 | 82.6 | −0.6 | −2.4 | 167.6 | varus | 147 | AD | 1 | 1 |
| F091 | 53 | F | B | 86.3 | 85.8 | −2.6 | −0.45 | 172.1 | neutral | 258 | AD | 1 | 2 |
| F092 | 59 | F | B | 85.5 | 86.5 | 1.8 | 1 | 172.0 | neutral | 258 | AD | 1 | 2 |
| F093 | 50 | F | B | 84.2 | 86.4 | 0.8 | 2.2 | 170.6 | valgus | 369 | AD | 1 | 3 |
| F094 | 50 | F | B | 86.7 | 86.5 | −2.1 | −0.2 | 173.2 | neutral | 258 | AD | 1 | 2 |
| F095 | 65 | F | B | 86.0 | 86.2 | −2.0 | 0.2 | 172.2 | neutral | 258 | AD | 1 | 2 |
| F096 | 67 | F | B | 91.3 | 85.5 | −6.9 | −5.8 | 176.8 | varus | 147 | AD | 1 | 1 |
| F097 | 52 | F | B | 84.7 | 88.1 | 0.0 | 3.4 | 172.8 | valgus | 369 | AD | 1 | 3 |
| F098 | 51 | F | B | 84.5 | 85.1 | 0.2 | 0.55 | 169.6 | neutral | 258 | AD | 1 | 2 |
| F099 | 56 | F | B | 82.3 | 87.4 | 5.1 | 5.1 | 169.7 | valgus | 369 | AD | 1 | 3 |
| F100 | 69 | F | B | 89.9 | 83.7 | −7.6 | −6.2 | 173.6 | varus | 147 | AD | 1 | 1 |
| F101 | 71 | F | C | 88.7 | 80.3 | −13.1 | −8.4 | 169.0 | varus | 147 | AD | 1 | 1 |
| F102 | 78 | F | C | 90.5 | 84.1 | −17.7 | −6.4 | 174.6 | varus | 147 | AD | 1 | 1 |
| F103 | 77 | F | C | 85.9 | 81.5 | −6.4 | −4.4 | 167.4 | varus | 147 | AD | 1 | 1 |
| F104 | 84 | F | C | 88.4 | 83.2 | −10.1 | −5.2 | 171.6 | varus | 147 | AD | 1 | 1 |
| F105 | 81 | F | C | 85.6 | 86.8 | −10.5 | 1.2 | 172.4 | neutral | 258 | AD | 1 | 2 |
| F106 | 69 | F | C | 85.5 | 87.4 | 3.0 | 1.9 | 172.9 | neutral | 258 | AD | 1 | 2 |
| F107 | 76 | F | C | 86.4 | 84.0 | −6.4 | −2.4 | 170.4 | varus | 147 | AD | 1 | 1 |
| F108 | 75 | F | C | 91.8 | 87.4 | −4.4 | −4.4 | 179.2 | varus | 147 | neutral | 2 | 4 |
| F109 | 85 | F | C | 86.4 | 84.9 | −6.4 | −1.5 | 171.3 | neutral | 258 | AD | 1 | 2 |
| F110 | 81 | F | C | 83.0 | 80.8 | −10.4 | −2.2 | 163.8 | varus | 147 | AD | 1 | 1 |

| | | | | | | | | | | | | | |
|---|---|---|---|---|---|---|---|---|---|---|---|---|---|
| F111 | 79 | F | C | 86.7 | 83.5 | −9.7 | −3.2 | 170.2 | varus | 147 | AD | 1 | 1 |
| F112 | 58 | F | C | 88.9 | 84.9 | 0.0 | −4.0 | 173.8 | varus | 147 | AD | 1 | 1 |
| F113 | 57 | F | C | 86.9 | 86.1 | −4.3 | −0.8 | 173.0 | neutral | 258 | AD | 1 | 2 |
| F114 | 86 | F | C | 86.8 | 85.6 | −3.6 | −1.2 | 172.4 | neutral | 258 | AD | 1 | 2 |
| F115 | 79 | F | C | 89.8 | 86.1 | −5.5 | −3.7 | 175.9 | varus | 147 | AD | 1 | 1 |
| F116 | 74 | F | C | 90.4 | 84.2 | −9.6 | −6.2 | 174.6 | varus | 147 | AD | 1 | 1 |
| F117 | 75 | F | C | 86.2 | 82.3 | −7.9 | −3.9 | 168.5 | varus | 147 | AD | 1 | 1 |
| F118 | 69 | F | C | 85.4 | 87.5 | −2.4 | 2.1 | 172.9 | valgus | 369 | AD | 1 | 3 |
| F119 | 77 | F | C | 90.8 | 84.9 | −16.3 | −5.9 | 175.7 | varus | 147 | AD | 1 | 1 |
| F120 | 84 | F | C | 87.7 | 87.1 | −6.8 | −0.6 | 174.8 | neutral | 258 | AD | 1 | 2 |
| F121 | 79 | F | C | 90.3 | 84.4 | −12.6 | −5.9 | 174.7 | varus | 147 | AD | 1 | 1 |
| F122 | 54 | F | C | 85.1 | 84.6 | −4.9 | −0.5 | 169.7 | neutral | 258 | AD | 1 | 2 |
| F123 | 74 | F | C | 88.2 | 81.5 | −11.7 | −6.7 | 169.7 | varus | 147 | AD | 1 | 1 |
| F124 | 58 | F | C | 87.5 | 81.3 | −12.8 | −6.2 | 168.8 | varus | 147 | AD | 1 | 1 |
| F125 | 68 | F | C | 89.0 | 82.1 | −18.2 | −6.9 | 171.1 | varus | 147 | AD | 1 | 1 |
| F126 | 73 | F | C | 90.5 | 84.4 | −15.9 | −6.1 | 174.9 | varus | 147 | AD | 1 | 1 |
| F127 | 79 | F | C | 91.1 | 84.7 | −9.7 | −6.4 | 175.8 | varus | 147 | AD | 1 | 1 |
| F128 | 79 | F | C | 90.0 | 88.8 | −8.8 | −1.2 | 178.8 | neutral | 258 | neutral | 2 | 5 |
| F129 | 72 | F | C | 86.7 | 85.8 | −3.7 | −0.9 | 172.5 | neutral | 258 | AD | 1 | 2 |
| F130 | 72 | F | C | 89.1 | 84.3 | −8.7 | −4.8 | 173.4 | varus | 147 | AD | 1 | 1 |
| F131 | 61 | F | C | 83.6 | 86.2 | 0.3 | 2.6 | 169.8 | valgus | 369 | AD | 1 | 3 |
| F132 | 58 | F | C | 87.0 | 85.3 | −2.9 | −1.7 | 172.3 | neutral | 258 | AD | 1 | 2 |
| F133 | 71 | F | C | 90.3 | 85.9 | −10.3 | −4.4 | 176.2 | varus | 147 | AD | 1 | 1 |
| F134 | 79 | F | C | 87.2 | 87.4 | −1.4 | 0.2 | 174.6 | neutral | 258 | AD | 1 | 2 |
| F135 | 72 | F | C | 88.0 | 86.1 | −7.3 | −1.9 | 174.1 | neutral | 258 | AD | 1 | 2 |
| F136 | 69 | F | C | 86.6 | 85.6 | −4.1 | −1 | 172.2 | neutral | 258 | AD | 1 | 2 |
| F137 | 62 | F | C | 87.7 | 83.6 | −6.1 | −4.1 | 171.3 | varus | 147 | AD | 1 | 1 |
| F138 | 72 | F | C | 90.8 | 87.6 | −6.9 | −3.2 | 178.4 | varus | 147 | neutral | 2 | 4 |
| F139 | 60 | F | C | 84.6 | 84.8 | −2.3 | 0.2 | 169.4 | neutral | 258 | AD | 1 | 2 |
| F140 | 66 | F | C | 87.5 | 92.0 | 1.7 | 4.5 | 179.5 | valgus | 369 | neutral | 2 | 6 |

| | | | | | | | | | | | | | |
|---|---|---|---|---|---|---|---|---|---|---|---|---|---|
| F141 | 66 | F | C | 89.5 | 83.7 | −9.5 | −5.8 | 173.2 | varus | 147 | AD | 1 | 1 |
| F142 | 75 | F | C | 89.0 | 84.1 | −14.0 | −4.9 | 173.1 | varus | 147 | AD | 1 | 1 |
| F143 | 72 | F | C | 86.1 | 85.1 | −6.7 | −1 | 171.2 | neutral | 258 | AD | 1 | 2 |
| F144 | 66 | F | C | 85.7 | 84.4 | −3.7 | −1.3 | 170.1 | neutral | 258 | AD | 1 | 2 |
| F145 | 69 | F | C | 87.7 | 84.1 | −6.0 | −3.6 | 171.8 | varus | 147 | AD | 1 | 1 |
| F146 | 74 | F | C | 88.3 | 86.5 | −3.1 | −1.8 | 174.8 | neutral | 258 | AD | 1 | 2 |
| F147 | 79 | F | C | 90.0 | 85.7 | −11.5 | −4.3 | 175.7 | varus | 147 | AD | 1 | 1 |
| F148 | 76 | F | C | 89.4 | 85.7 | −5.8 | −3.7 | 175.1 | varus | 147 | AD | 1 | 1 |
| F149 | 88 | F | C | 93.2 | 86.6 | −12.9 | −6.6 | 179.8 | varus | 147 | neutral | 2 | 4 |
| F150 | 71 | F | C | 92.1 | 86.3 | −3.8 | −5.8 | 178.4 | varus | 147 | neutral | 2 | 4 |
